# Observed decrease in light precipitation in part due to urbanization

**DOI:** 10.1038/s41598-022-07897-8

**Published:** 2022-03-09

**Authors:** Suonam Kealdrup Tysa, Guoyu Ren

**Affiliations:** 1 Department of Atmospheric Science, School of Environmental Studies, China University of Geosciences (CUG), Wuhan, China; 2 Laboratory for Climate Studies, National Climate Center, China Meteorological Administration (CMA), Beijing, China

**Keywords:** Climate change, Hydrology

## Abstract

Decrease in light precipitation (LP) frequency has been reported in many regions. However, reason for the decrease remains poorly understood. Here, we quantify urbanization effect on LP (< 3.0 mm day^−1^) trend in China over the period 1960–2018. We show that urbanization has significantly affected the decreasing LP trend. The urbanization effect becomes more significant as the definition of LP becomes stricter, with the largest effect appearing in trace precipitation change (< 0.3 mm day^−1^) (LP_0.3_) during summer and autumn. We estimate that at least 25% of the decreases in LP_0.3_ days and amount are due to urbanization near the observational stations. Our analysis thus confirms that urbanization has largely contributed to the observed downward trend in LP, and the large-scale change in LP is less than previously believed.

## Introduction

The observed decrease in Light Precipitation (LP) frequency in China over recent decades has been well documented^1–5^. This is consistent with similar decreasing trends reported in many regions globally^6,7^. The downward trend of LP is mainly due to changes in the extremely light precipitation events (0.1–1.0 mm/day) in China, with a rapidly decrease occurring after 1982^5^. Since LP has a far higher frequency than other types of precipitation, a decrease in LP frequency causes a significant drop in the total precipitation days and a significant increase in the daily mean intensity of precipitation^8^.

Studies have attributed the LP reduction to the following factors: (i) The indirect effect of aerosols. An increase in aerosols acting as cloud condensation nuclei leads to an increase in the cloud droplet number concentration and a decrease in droplet size, with an assumption of an invariable cloud liquid water path^9^. Thus the impact of aerosols on LP or warm-rain process leads to increased cloud lifetime and decreased raindrop concentration^4,10^. Recent studies^11,12^ also assented that light and low-intensity rainfalls appears to be influenced by local anthropogenic influences through changes in cloud microphysical processes in China. (ii) Temperature effect. Due to the increasing temperature in the lower troposphere, a rise in saturation vapour pressure suppresses vapour condensation and leads to a decrease in relative humidity (RH), inducing a reduction in LP^13,14^. The reduction of RH in the boundary layer also causes the re-evaporation of small raindrop^15^. (iii) Atmospheric static stability. One study links the decrease in LP to the atmospheric static stability associated with global warming^6,16^, since enhancement of upward motion leads to a decrease in atmospheric stability, resulting in a reduction in LP.

However, it is unclear which factors are crucial in the widespread decrease in LP frequency, and as a local anthropogenic factor, whether or not the urbanization process has exerted an influence on the observed change in LP.

China has experienced rapid urbanization in the past several decades, which has had increasing impacts on the local climate around observational stations^17–19^. A marked urban–rural contrast appears in the influential factors affecting LP in the country: (i) Abundant particulate pollutants in urban areas are an important source of aerosols^20^, and more severe air pollution in urban areas than rural areas is observed^21^. (ii) Strong urban heat island (UHI) effect has been observed at many urban meteorological stations. The increasing UHI intensity has contributed significantly to the reported warming trend estimated from national stations in China^15,17^. (iii) Urbanization has also impacted near-surface atmospheric moisture^22^, and the urban dryness island (UDI)^23^ effect has been significantly intensified by rapid urban land expansion^24,25^. Combined with other urban drivers, the urbanization process may have affected the trend of observed LP. It is thus interesting to examine if the urbanization has affected the long-term change in LP in China, in order to better understand the change over a sufficiently large region. The result would also have great practical significance for assessing the impact of precipitation change on ecosystems, agriculture, and water resource management due to the high infiltration efficiency of LP in the soil.

Intriguingly, the decrease in LP frequency obtained through climate model simulation is not as large as that reported from observations^26,27^. The underestimation of the negative LP trend has been regarded as the primary deficiency of the current models, and most researchers believe that it may be associated with improper processing of the cloud and aerosol effects in the climate models^28,29^. Understanding the characteristics and causes of the observed decrease in LP will therefore facilitate the evaluation and improvement of climate models, which could then be more confidently used in studies of climate and climate change.

We evaluate here the urbanization effect on and its contribution to the estimated trends of LP days and amount based on a frequently applied dataset of observational station network in China. To do this, we apply an urban–rural method^17,19^ to examine the trend differences between national stations and nearby rural stations, and regard the difference as an indicator of the urbanization effect on the long-term trend of LP at the national stations, with the assumption that the linear trends of any two observational stations within a region are the same under the influence of global and regional drivers. A vital issue for this procedure to be effective is the determination of rural stations as benchmarks^15^.

In our previous classification of meteorological stations with different urbanization levels, a dataset of rural station network in China (Supplementary Fig. 1b,c) is established^17^. The rural station network has been affirmed to be applicable in study of the urbanization effect on observational surface air temperature (SAT) trend^17^. Applying the daily precipitation data of the rural stations and all of the national stations, we analyses the urbanization effect on and its contribution to the trends of days and amounts of LP at the national stations in China (see “Methods” and Supplementary data for details). The LP here is defined as daily precipitation less than 3.0 mm, which is further classified into three different levels of less than 0.3 mm (LP_0.3_), 1.0 mm (LP_1.0_), and 3.0 mm (LP_3.0_), per day, respectively. The days and amount of LP_0.3_, LP_1.0,_ and LP_3.0_ are abbreviated as DLP_0.3_, DLP_1.0_ and DLP_3.0_, and ALP_0.3_, ALP_1.0_ and ALP_3.0_, respectively (Supplementary data for details).

## Results

### Changes of LP

Significant reductions in the total precipitation days and amount are detected only for precipitation lower than the 20th percentile of daily precipitation at each station during 1960–2018 (Supplementary Fig. 2a,b). The trends are especially evident for precipitation lower than the 10th percentile. Decreasing trends are observed in most parts of China (Supplementary Fig. 3), particularly in the east China (EC), central China (CC) and southern southwest China (sSWC) (Supplementary Fig. 3c,d). No significant decrease in intensity is observed (Supplementary Fig. 2c). Thus, the possible urbanization effects on the changes in only days and amount of LP (DLP and ALP) are further examined.

**Figure 1:**
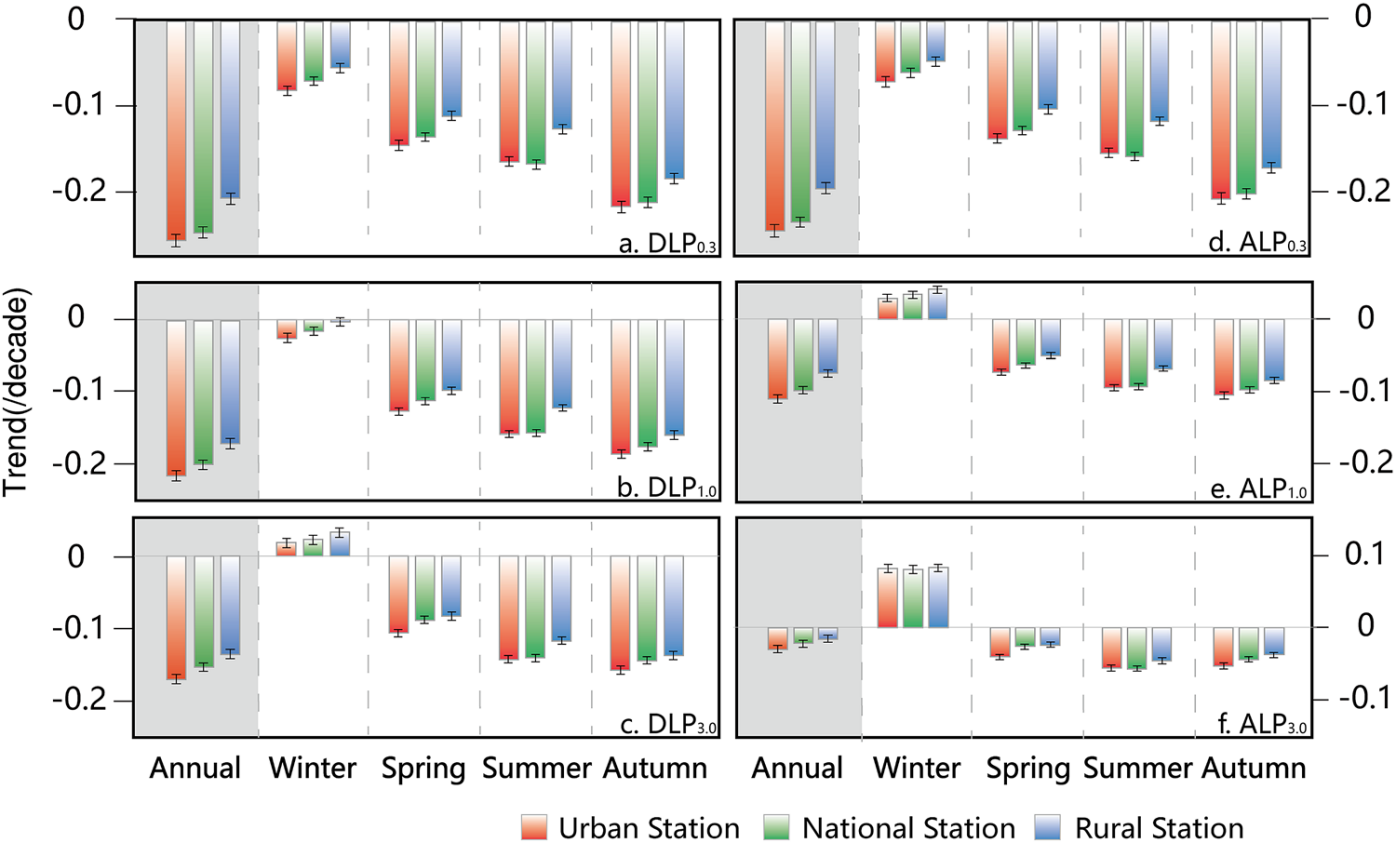
Comparison of changes in observed light precipitation (LP) for different station networks. The linear trends of the standardized anomalies of the annual and seasonal total DLP (DLP_0.3_: **a**; DLP_1.0_: **b**; DLP_3.0_: **c**) and ALP (ALP_0.3_: **d**; ALP_1.0_: **e**; ALP_3.0_: **f**) at the national stations (green), urban stations (red) and rural stations (blue) in China during the period of 1960–2018, along with their standard errors. Units: decade^−1^.

Significant decreases in all of the LP metrics are observed at the national stations during 1960–2018 (Fig. 1). The most significant negative trends appear in autumn and summer, and the smallest reduction occurs in winter. In addition, as the definition of LP becomes stricter, the reduction in DLP and ALP becomes larger. A more significant decrease in DLP than ALP is seeable, although the magnitudes of change in ALP_0.3_ and DLP_0.3_ are comparable. It is also notable that a more significant decrease in LP is detected at the urban stations than at the national and rural stations in China (Fig. 1), and this difference is more apparent for LP_0.3_. The linear trends of the standardized anomalies of the annual total DLP_0.3_ and ALP_0.3_ at the urban, national, and rural stations are − 0.254, − 0.244 and − 0.205 decade^−1^, and − 0.243, − 0.234 and − 0.194 decade^−1^, respectively, indicating a cascading drop in absolute values from more urbanized to more rural observational networks.

### Urbanization effect and its contribution on LP change

**Figure 2:**
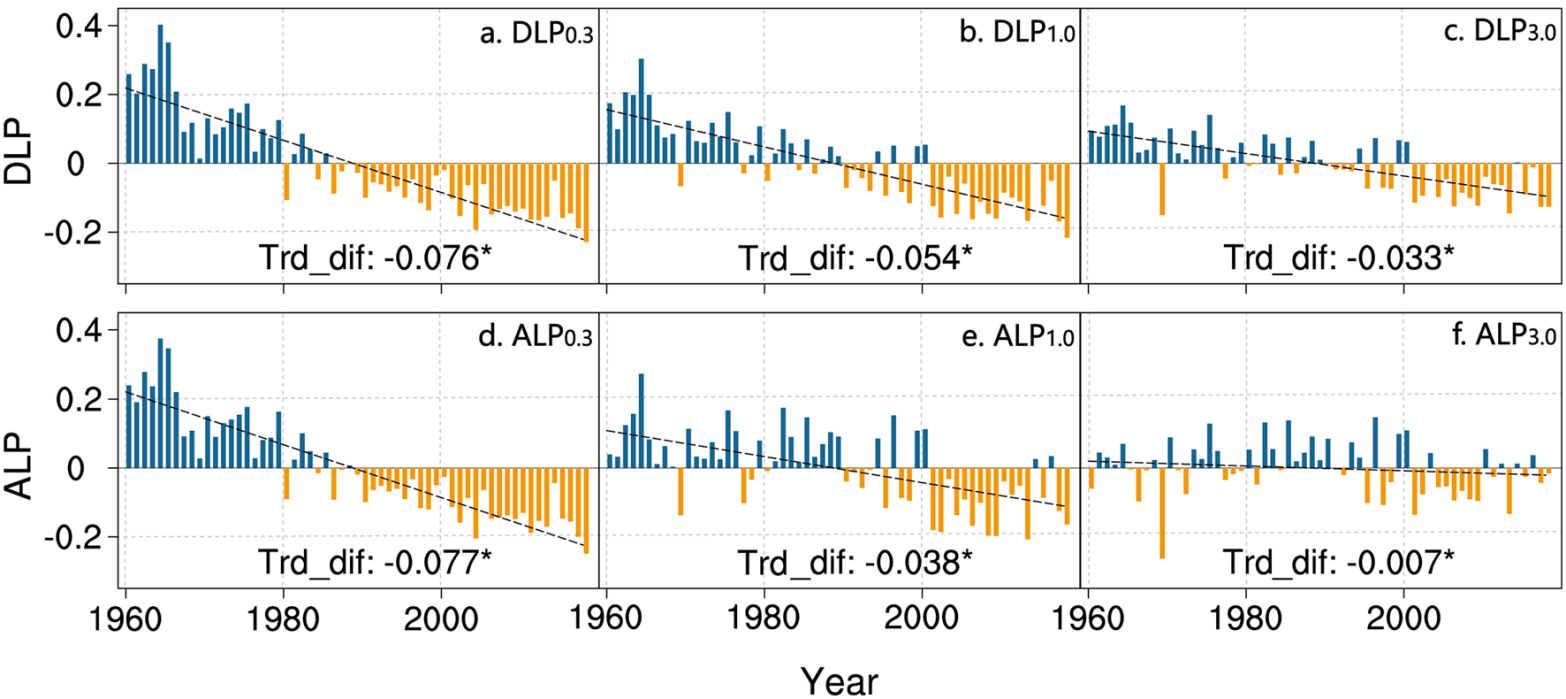
Urbanization effect on light precipitation (LP) change for the national station network. Shown in this figure are **t**he difference time series of the standardized anomalies of the annual total DLP (**a** DLP_0.3_; **b** DLP_1.0_; **c** DLP_3.0_) and ALP (**d** ALP_0.3_; **e** ALP_1.0_; **f** ALP_3.0_) between national and rural stations in China during the period of 1960–2018. Trd_dif: the linear trend of the difference series (units: decade^−1^); asterisks: statistically significant trends at the 95% confidence level.

**Figure 3:**
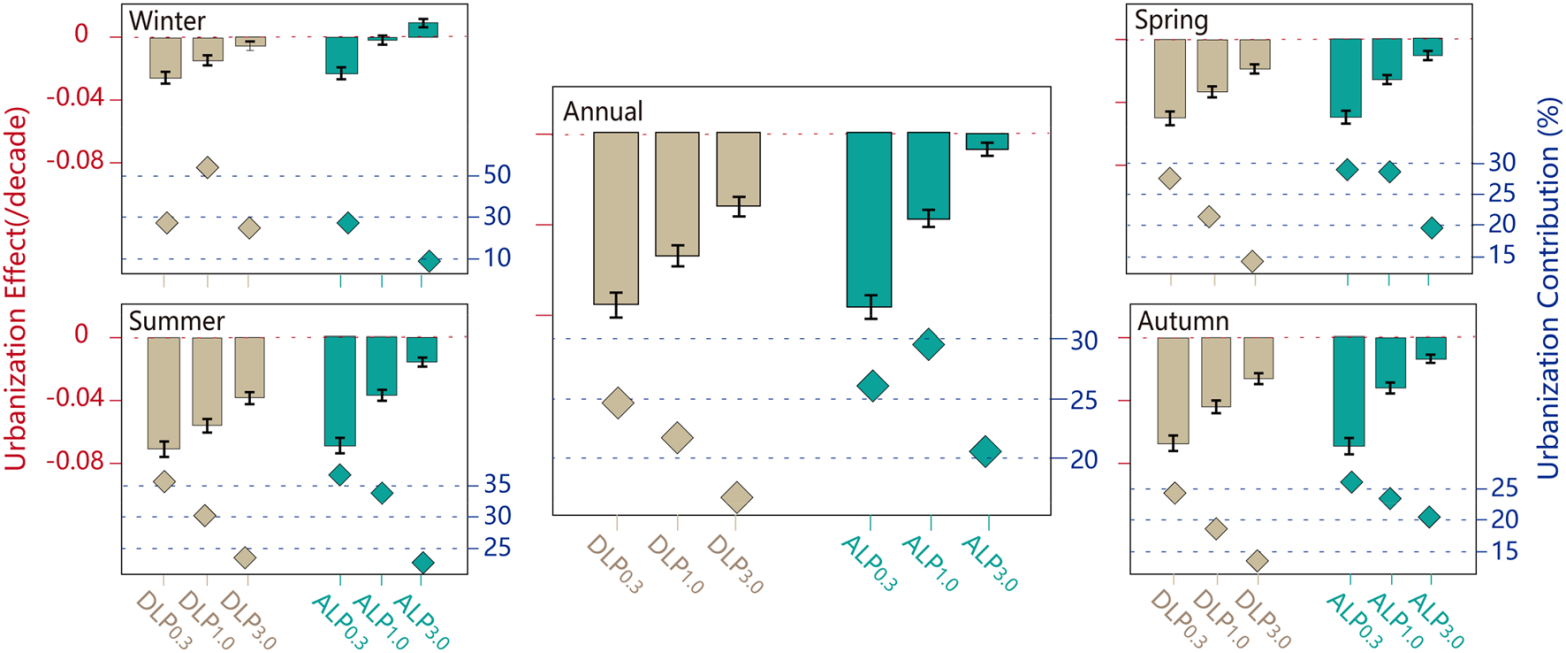
Urbanization effect on and its contribution to light precipitation (LP) change for the national station network. The urbanization effects (bar chart, units: decade^−1^) and its contributions (scatter plot, units: %) to the trends of the standardized anomalies of the annual and seasonal total DLP and ALP at the national stations in China during the period of 1960–2018, along with urbanization effects’ standard errors.

Urbanization effects can be clearly observed in the decreasing DLP and ALP at the national stations (Figs. 2, 3), which are statistically significant at the 95% confidence level. In addition, as the definition of LP becomes stricter, the urbanization effect on the annual DLP and ALP change becomes larger. It is also more obvious for the change in DLP than in ALP when LP is defined by different standards, although the decreases in ALP_0.3_ and DLP_0.3_ are comparable. The urbanization effects on the standardized anomalies of the annual DLP_0.3_ and ALP_0.3_ changes at the national stations are − 0.076 decade^−1^ and − 0.077 decade^−1^, respectively. In terms of seasonal changes, all urbanization effects showed significant downward trends (Fig. 3, Supplementary Figs. 4, 5) except for winter ALP_1.0_. The largest negative urbanization effects on LP_0.3_ changes appear in summer and autumn. For the LP_1.0_ and LP_3.0_, a more significant urbanization effect occurs in the summer DLP change. Although the winter DLP_3.0_ has increased at the national stations (Fig. 1c), urbanization has significantly reduced the upward trend (Fig. 3, Supplementary Fig. 4c).

Figure 3 also shows the urbanization contributions to the observed decreasing trends of various LP categories. Similarly, as the LP gets lighter, the urbanization contribution to DLP reduction becomes more significant, except in winter. The annual urbanization contributions are 24.8% and 26.0%, and the seasonal urbanization contributions in summer even reach 35.7% and 36.9%, for the DLP_0.3_ and ALP_0.3_ changes, respectively. The largest urbanization contributions are observed in winter (54.9% and 25.1%, respectively) for DLP_1.0_ and DLP_3.0_. For ALP, a large urbanization contribution is observed for ALP_1.0_ (29.5%), reaching 33.9% in summer. The urbanization contribution to the winter ALP_1.0_ change is not analysed since the urbanization effect is not statistically significant (Fig. 3, Supplementary Fig. 5b).

**Figure 4:**
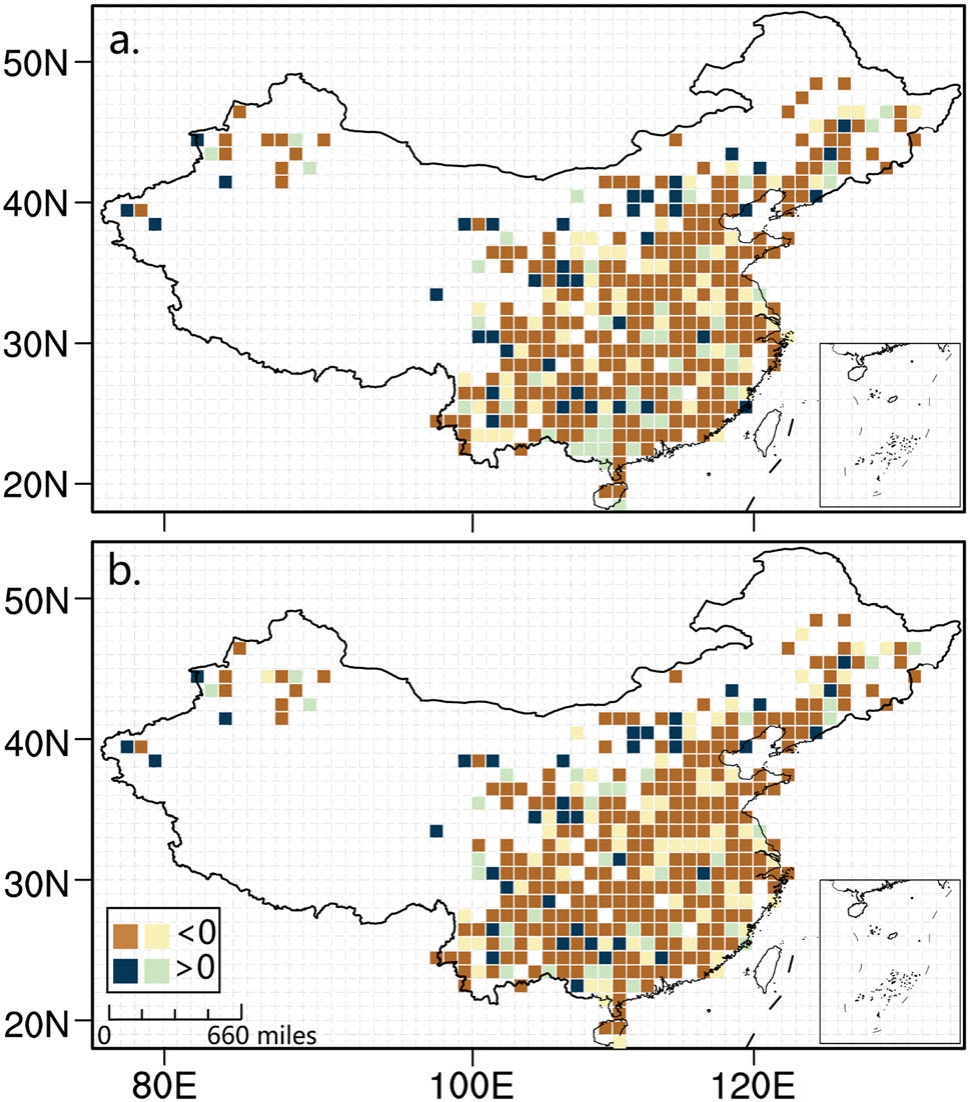
Spatial distribution of urbanization effect on light precipitation (LP) change for the national station network. Spatial distributions of urbanization effects on the trends of the standardized anomalies of the annual total DLP_0.3_ (**a**) and ALP_0.3_ (**b**) at the national stations in China during the period of 1960–2018. Grid size: 1°×1° latitude and longitude; units: decade^−1^; dark brown/blue grids: statistically significant at the 95% confidence level. NCAR Command Language (NCL) (Version 6.4.0; https://www.ncl.ucar.edu) was used to create the maps.

Figure 4 shows the spatial distribution of the urbanization effect on the annual LP_0.3_ change at the national stations. DLP_0.3_ and ALP_0.3_ have experienced a significant urbanization effect in most regions, especially in north China (NC), EC and CC (Supplementary Fig. 6), which correspond to the regions where LP decreases more significantly (Supplementary Fig. 3c,d) and the most rapid urbanization in China has happened over the last half a century. In summer, the regions with remarkable negative urbanization effects are seen in NC and EC (Supplementary Figs. 7c,g, 8c,g). The negative urbanization effect on the autumn LP_0.3_ trend is mainly distributed in CC, EC and sSWC (Supplementary Figs. 7d,h, 8d,h). EC, CC and south China (SC) have also witnessed a great negative urbanization effect on the spring LP_0.3_ trends (Supplementary Figs. 7b,f, 8b,f).

The urbanization effects on LP_0.3_ changes at the national stations over three super-city clusters [Beijing–Tianjin–Hebei (BTH), Yangtze River Delta (YRD), and Pearl River Delta (PRD)] (green box in Supplementary Fig. 9) are more significant than those over China on a whole (Supplementary Table 1). Comparable urbanization effects on and contributions to DLP_0.3_ and ALP_0.3_ trends are found in each of the three regions, and an urbanization contribution of up to 56.5% (52.8%) to the annual DLP_0.3_ (ALP_0.3_) trend can be seen in the BTH region. Furthermore, in the BTH and PRD regions, more significant negative urbanization effect on the seasonal LP_0.3_ appears in summer and autumn.

## Discussion

**Figure 5:**
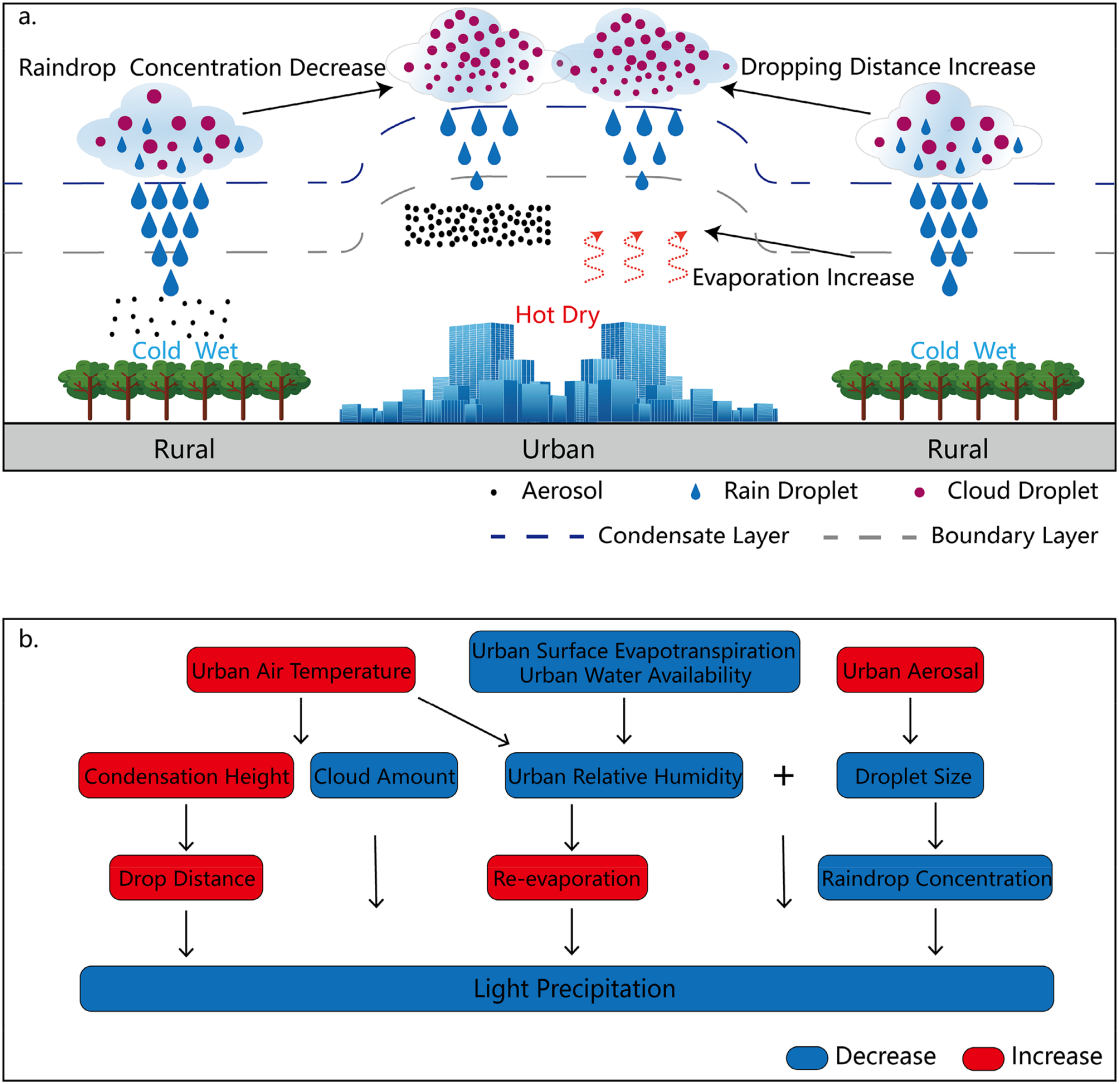
A schematic diagram of the possible impacts of urbanization on LP change.

The physical causes of the significant urbanization effect on the LP change are understandable. Urban air pollution, the UHI effect, and the UDI effect around the observational stations are important factors (Fig. 5). Previous studies show that there is an urban–rural contrast in air pollution, with a higher concentration of aerosols in urban areas than in rural areas^21,30^. Our analysis also shows that urbanization has resulted in an increasing aerosol optical depth (AOD) at the national stations during the past 39 years (Supplementary Figs. 10b, 11c). Moreover, the stations with larger urban–rural contrast of AOD trends exhibit stronger urbanization effects on DLP_0.3_ trends (Supplementary Fig. 11e). Aerosols act as cloud condensation nuclei, and their increase may have led to an increase in the cloud droplet concentration and a decrease in the droplet size, resulting in a reduction in the raindrop concentration, LP frequency and amount^31–33^ near urban stations under conditions of insufficient atmospheric moisture. Thus, the more significant and faster AOD increases over urban areas than rural areas may have been one of the potential causes of the observed urbanization effect on the LP trend at the national stations in the country.

The UHI and UDI effects are well-recognized urban climatic phenomena, and their enhancement with urbanization^24,25^ may have exerted an influence on the LP decrease observed at the national stations. A higher air temperature increases the height of condensation and the cloud base over urban areas by raising the saturation vapour pressure. This leads to a lower chance for small raindrops to precipitate onto the ground due to the increased travel distance^34^, inducing a reduction in LP. Large-scale warming has the same influence on LP, which may be one of the reasons that rural stations have also experienced a significant reduction in LP. In addition, the UHI effect reduces the RH in the canopy layer and boundary layer of urban areas^23^. In combination with the less surface evaporation^35^, this facilitates the formation of the UDI effect, resulting in a higher chance for small raindrops to re-evaporate before they reach the ground. Urbanization has resulted in a significant decrease in RH at the national stations during the past 39 years (Supplementary Fig. 11d), and the stations with greater urban–rural contrast of relative humidity trends also exhibit greater urbanization effects on DLP_0.3_ trends (Supplementary Fig. 11f).

As a systematic measurement error, wind-induced under-catch should be removed from the recorded LP data to obtain an accurate estimation of precipitation change^36,37^. Larger near-surface wind speed (SWS) will result in lower catch rates of rain gauge, especially for trace precipitation. As a special form of land use and land cover change, urbanization is positively correlated with surface friction, which is one of the primary determining factors of the observed “stilling” (a reduction in the observed SWS)^38–41^. Previous studies pointed out that the observed SWS decline may be mainly due to the underlying surface changes caused by urbanization around observational stations in China^42,43^. The decreased urban SWS should have reduced the under-catch and the underestimation of LP at the national stations relative to the rural stations, resulting in an increase in LP frequency and amount at the national and urban stations. Obviously, this contradicts what has been observed. Thus, the decreasing LP at the national stations should be larger and more significant when the possible impact of the urban SWS change is considered. Therefore, the impact of urbanization on the SWS decline may have offset to some extent the urbanization contribution to the decrease in LP estimated in this study.

Our analysis helps to understand the structure and causes of long-term precipitation changes observed at the national stations in China. In addition, the findings reported here are also significant in terms of the two following aspects:

1. Observational bias of precipitation change. Previous studies regarded the decrease in LP frequency as a large-scale change in global land. Our analysis shows that it partially originates from an observational bias at the current meteorological stations primarily located in or near urban areas. Compared to urban areas that account for only approximately 1–2% of the total land area of China^15^, the large areas other than urban districts have not registered a similar magnitude of LP decrease over the last decades. This difference is important because assessments of the climate change impacts on water resources, agriculture, and ecosystems have to be made in regions outside of urban extents, and the previous decreasing LP trends on a regional scale have been significantly overestimated. The LP frequency may also be decreasing in large rural areas and wildlands, but the decrease must not be as large as previously reported.
2. Performance of models in simulating precipitation change. Climate models are unable to accurately simulate the structure of large-scale precipitation changes. In particular, the modelled LP decrease is considered small compared to that based on observations^26,27^. This inconsistency is the so-called “drizzle effect”. Researchers believe that there is a need to deepen the understanding of the physical and chemical processes of clouds and aerosols^27,28^ and to improve the convective parameterization scheme of the models^29^. In carrying out model downscaling analysis, some studies attempt to correct the model bias to obtain a “more reasonable” frequency and amount of LP events^44,45^. However, our findings in this study shows that the “drizzle effect” may have been exaggerated because of the observational bias in the current precipitation data. The performance of the models is not as bad as we previously believed.

In any case, our analysis indicates an urgent need to examine the influence of local anthropogenic land-use change (urbanization) on precipitation observations, and to develop a set of methods to correct the observational bias in the currently applied historical precipitation data, if we intend to robustly detect and model large-scale changes in varying levels of precipitation, including LP.

## Conclusions

We can therefore draw the following conclusions:

(i) More significant decrease in light precipitation (< 3.0 mm/day) is detected at the national stations than at the rural stations in China during 1960–2018. Urbanization significantly affected the decreasing trend. The greatest urbanization effect is observed on the change of trace precipitation (< 0.3 mm/day), and this phenomenon is more obvious in summer and autumn.
(ii) Changes in light precipitation in particular in trace precipitation have experienced a significant urbanization effect at most national stations, especially in north China, east China and central China, which well correspond to the regions where the most rapid urbanization has happened over the last half a century.
(iii) The urbanization contributions to the declines of days and amount of trace precipitation reach 24.8% and 26.0%, respectively, in China as a whole during 1960–2018, with the largest contribution occurring in summer (35.7% and 36.9%, respectively) in seasons and Beijing-Tianjin-Hebei areas (> 50%) in case regions.
(iv) The worsened air pollution, and the heightened intensity of urban heat island and urban dryness island in urban areas compared to the rural areas might have been the main factors causing the significant urbanization effect on the long-term downward trend of light precipitation at the observational stations of the country during the last decades.

## Methods

### Urbanization indicator

Our previous study^17^ found that the proportionality of urban land use in different circumferences around the observational station can be used as an indicator of the comprehensive urbanization level, and it can be applied to determine the rural station.

The land use/land cover dataset with a 1.0 km spatial resolution in 2015 was provided by the Resources and Environmental Sciences Data Center, Chinese Academy of Sciences (https://www.resdc.cn/data.aspx?DATAID=184). The built-up areas of large, medium, small cities, towns, and other construction lands are used to categorize urban land use.

We extracted and calculated the proportionality of urban land use in buffer circles with radii of 1.0 km to 16.0 km around all 2419 observational national stations, which means that the possible impacts of urban land use on the observational variable of the station in varied spatial extents from microscale to mesoscale are considered^17^. Then, based on the maximum and the area‐weighted average of the proportionality of urban land use in the 16 buffer circles around the station, all national stations were classified into six categories, including U1, U2, U3, U4, U5, and U6, in order of enhancement of urbanization level. This categorization assumes that U1 is hardly affected by urbanization, and the other stations are affected by lower (U2), low (U3), medium (U4), high (U5), and higher (U6) urbanization levels. See Tysa et al.^17^ for details.

### Rural station network

In our previous study, the rural station network in China was established by assigning at least one rural station in each 2.0°×2.0° grid, which was used to estimate the impact of urbanization on the trend of the regional SAT series. That is, based on several specific criteria, the first and second categories of stations (U1 and U2) were applied as rural stations to form a rural station network. U2 stations are used because there are no U1 stations in most grids in NC, EC, CC and SC, due to the significant urbanization in these areas.

Since the regional differences in precipitation are greater than those in SAT, the rural station network with a greater spatial resolution of 1.0°*1.0° is established in this study according to the following criteria^15^: (i) observations with a sufficient data series length and good continuity (i.e., stations with records beginning no later than 1960), (ii) high stability and limited relocation of observational sites (i.e., stations relocated less than three times after 1960), (iii) immunity to urbanization influences (i.e., stations with the lowest urbanization level in each 1.0°×1.0° grid). The new rural station network is shown in Supplementary Fig. 1b, where the highest urbanization level of the rural station is U3, with such stations distribute mainly in the NC and EC. The numbers of U1, U2, and U3 stations in the rural station network are 262, 265, and 324, respectively (Supplementary Fig. 1c). Finally, the remaining stations in each grid were determined to be urban stations, which reached a total number of 1569 (Supplementary Fig. 1d).

The national stations, urban stations, and rural stations are thus composed of all, urban, and rural stations in China, respectively, in this study.

A key issue in assessing the urbanization effect is the determination of the rural stations. The rural stations utilized in this study are the stations with the lowest urbanization level in each grid. However, most of them are not actual rural stations, and the U2 and U3 stations are actually located in or near small cities or towns. Therefore, the rural LP series itself may have been affected by urbanization to a certain extent, although the effect is expected to be substantially smaller than that in the urban and national LP series. Therefore, the estimated effects and contributions of urbanization given in this paper should be regarded as conservative.

### Definition, calculation and assumption

In this study, the annual and seasonal total DLP and ALP are the number of LP days and sum of the amount of LP, and the annual mean AOD values are the 12-month average. The four seasons are divided according to climatological seasons: spring (March–May), summer (June–August), autumn (September–November), and winter (December–February).

The national, urban, and rural time series in each grid are established by averaging the values of national stations, urban stations, and rural stations. Then, the national, urban, and rural time series in the study region, subregion, and case region are established by area-averaging the national, urban, and rural series in all grids in the study, respectively.

The linear trend of the difference series between the national series and rural series is defined as the urbanization effect. The percent proportion of the urbanization effect on the overall trend is defined as the urbanization contribution. This value is calculated if the urbanization effect is statistically significant at a 95% confidence level.

The urbanization effect is defined as follows:

1 $$UE = T_{{\text{n}}} - T_{r}$$

The urbanization contribution is defined as follows:

2 $$UC = \left| {\frac{{T_{{\text{n}}} - T_{r} }}{{T_{n} }}} \right| \times 100\%$$

where T_n_ and T_r_ are the linear trends of the national and rural LP series, respectively.

An assumption behind the procedure is that the background trends of climatic variables including light precipitation are the same within a small area with a radius less than 100–200 km. The trend difference of the climatic variables is thus attributed to a locally natural and anthropogenic factor. The assumption has been proved valid in previous studies on urbanization effect in the SAT data series of urban and national stations in China^15,17,46^. Here we applied this assumption to the evaluation of urbanization effect on LP change in China.

The linear trend of the time series is estimated by the ordinary least squares method. The statistical significance of the linear trend was judged by Student’s t‐test. The trend of the series is considered statistically significant if it passes the 95% confidence level. In addition, all calculations of LP time series are based on their standardized anomalies in this study. Standardized anomaly is the ratio of LP anomaly to standard deviation, and LP anomaly is the departure of LP from its mean during the study period (1960–2018).

## Supplementary Information

Supplementary Information.
