# Supplementary material for: Observed decrease in light precipitation in part due to urbanization
Source: Sci Rep. 2022 Mar 9;12:3864. doi: 10.1038/s41598-022-07897-8 (PMC8907232; doi:10.1038/s41598-022-07897-8)
Supplement: Supplementary file 1 — Supplementary Information. [file 41598_2022_7897_MOESM1_ESM.doc]

**Observed decrease in light precipitation in part due to urbanization**

**SUPPLEMENTARY INFORMATION**

Suonam Kealdrup Tysa^1^ Guoyu Ren^1, 2, *^

^1^ Department of Atmospheric Science, School of Environmental Studies, China University of Geosciences (CUG), Wuhan, China.

^2^ Laboratory for Climate Studies, National Climate Center, China Meteorological Administration (CMA), Beijing, China

^*^Email: [guoyoo@cma.cn](mailto:guoyoo@cma.cn)

# Data and region

Long-term and gauge-based daily precipitation data are utilized in this study and are from the “China National Surface Meteorological Station Homogenization Precipitation Monthly Dataset (V1.0)” provided by the National Meteorological Information Center, China Meteorological Administration (NMIC, CMA). As the dataset from the highest spatial density national observational network consisting of 2,419 stations (Supplementary Fig. 1a), the dataset has undergone strict quality control, including tests of the climatological limits, internal and spatial consistency. Moreover, the homogeneity test and correction of the precipitation series have also been performed by applying the standard normal homogeneity test (SNHT)^1^. The dataset is extensively used to investigate the characteristics of precipitation change in China ^2^. The problem of missing data is processed by discarding year values with more than three missing monthly records, season values with more than one missing monthly record, and annual and seasonal series with more than five missing years during the study period. In this study, the study period is 1960-2018.

Since LP is generally defined as less than the 50th percentile of daily precipitation^3^, and we found that the 10th, 20th, 30th, 40th and 50th percentile of daily precipitation at 99.5% of stations are 0.3, 1.0, 2.0, 3.0 and 5.0 mm day^-1^ in China, respectively. Furthermore, similar results are obtained in our experiments when the LP is defined as lower than 2.0 and 3.0 mm day^-1^, and a lower urbanization effect is obtained when the LP is defined as lower than 5.0 mm day^-1^. Thus, three types of LPs are defined in this study, including LP_0.3_, LP_1.0_, and LP_3.0_, which represent daily precipitation less than 0.3 mm, 1.0 mm, and 3.0 mm, respectively. In this paper, the days and amount of LP_0.3_, LP_1.0_, and LP_3.0_ are abbreviated DLP_0.3_, DLP_1.0_, DLP_3.0_, ALP_0.3_, ALP_1.0_, and ALP_3.0_, respectively.

The monthly AOD product with a spatial resolution of 0.625°*0.5° ^4^ during the period of 1980-2018 is used, which is from MERRA-2 (the second Modern-Era Retrospective analysis for Research and Applications). In addition, by extracting the grid AOD value of each station, the AOD series of each station is established.

Eight subregions are divided in China (Supplementary Fig. 9a), referring to Chang et al.^5^. The subregions include northeast China (NEC), north China (NC), northwest China (NWC), east China (EC), central China (CC), northern southwest China (nSWC), southern southwest China (sSWC), and south China (SC). Three super-city clusters in China (Supplementary Fig. 9b-d) – Beijing-Tianjin-Hebei (BTH), Yangtze River Delta (YRD), and Pearl River Delta (PRD) – are selected as case regions to examine the urbanization effects and contributions. The boundaries of these three regions are based on the global urban boundaries from the global artificial impervious area (GAIA) data from 2018; this dataset has a spatial resolution of 30 m. A grid size of 1.0°*1.0° is used within the urban boundaries (Supplementary Fig. 9a).

# References

# Supplementary Figures and Table


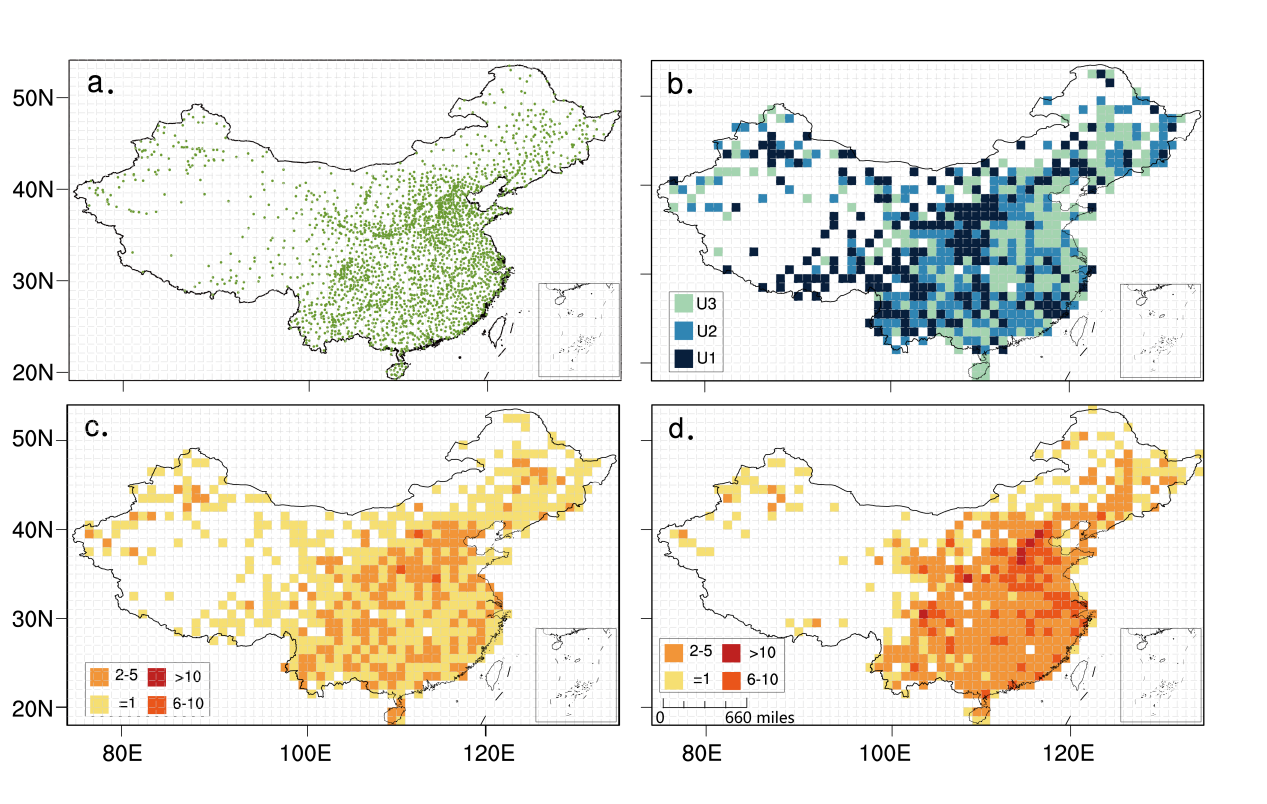


**Supplementary Figure 1.** Distribution of stations for the national station network **(a)**, the grid boxes with the urbanization levels of rural stations **(b)**, the grid boxes with the number of rural stations **(c)** and the grid boxes with the number of urban stations **(d)** in China. Grid size: 1.0°*1.0° latitude and longitude. NCAR Command Language (NCL) (Version 6.4.0; https://www.ncl.ucar.edu) was used to create the maps.


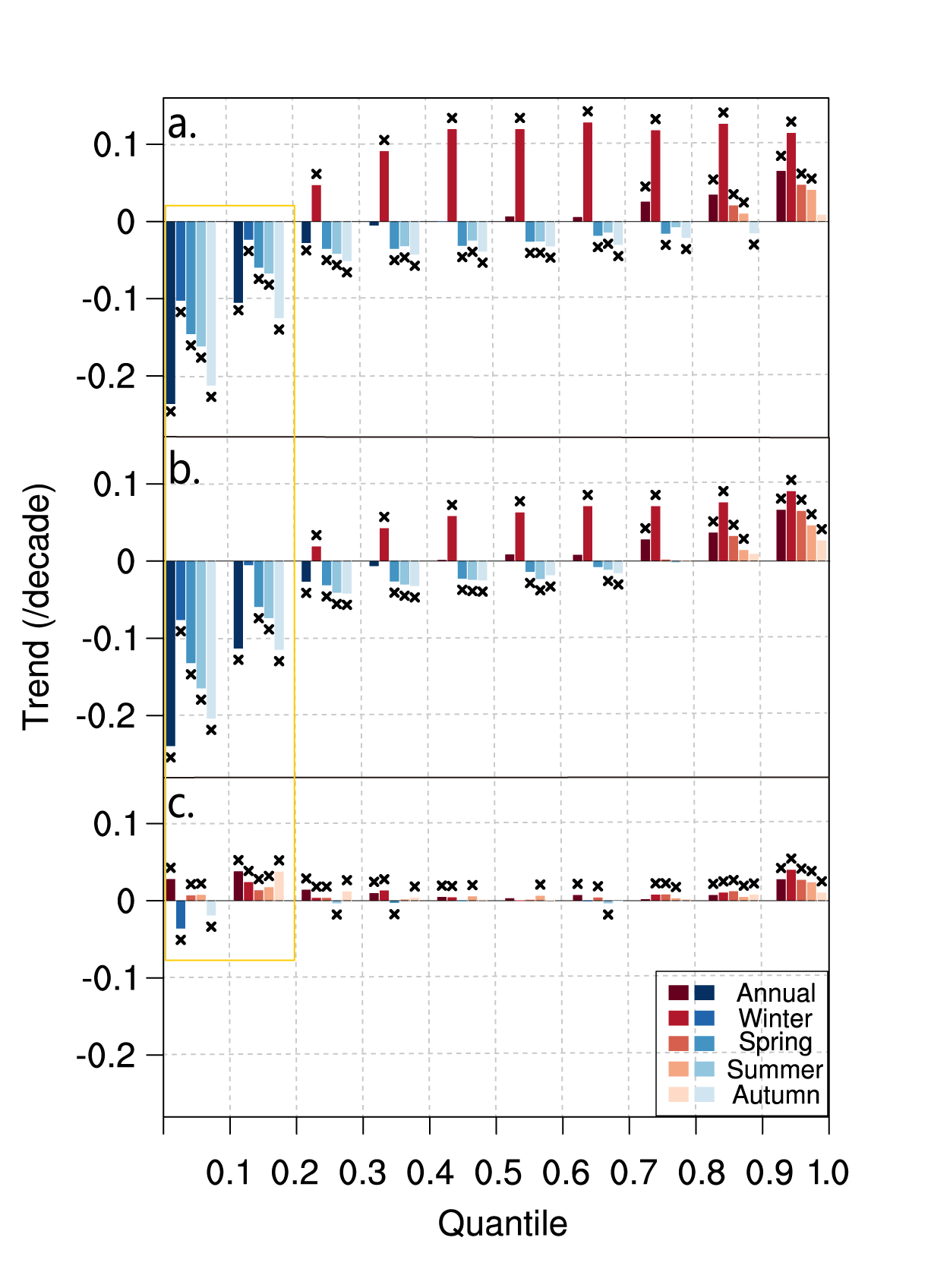


**Supplementary Figure 2.** Linear trends of the standardized anomalies of the annual and seasonal total precipitation amount **(a)**, total precipitation days **(b)**, and mean precipitation intensity **(c)** in different quantiles (0-0.1, 0.1-0.2, ..., 0.9-1.0) at the national stations in China during the period of 1960-2018. Units: decade^-1^; asterisks: statistically significant trends at the 95% confidence level.


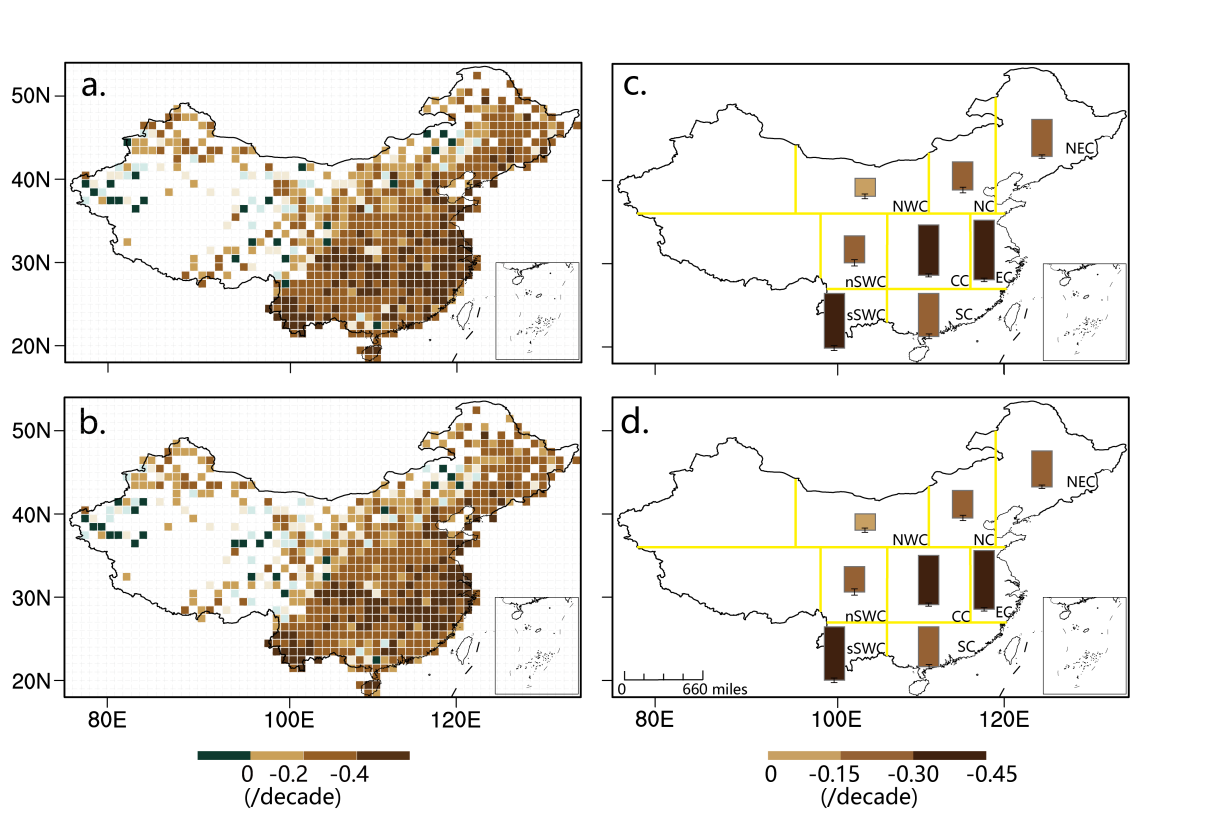


**Supplementary Figure 3.** Spatial distributions of the trends of the standardized anomalies of the annual total precipitation days **(a)** and amount **(b)** at the national stations in China during the period of 1960-2018, only for precipitation lower than the 10th percentile of daily precipitation at each station. Grid size: 1.0°*1.0° latitude and longitude; units: decade^-1^; light brown/green grids: statistically insignificant at the 95% confidence level. Mean trends of the standardized anomalies of the annual total precipitation days **(c)** and amount **(d)** at the national stations over eight subregions, along with their standard errors, only for precipitation lower than the 10th percentile of daily precipitation at each station. NCAR Command Language (NCL) (Version 6.4.0; https://www.ncl.ucar.edu) was used to create the maps. Units: decade^-1^


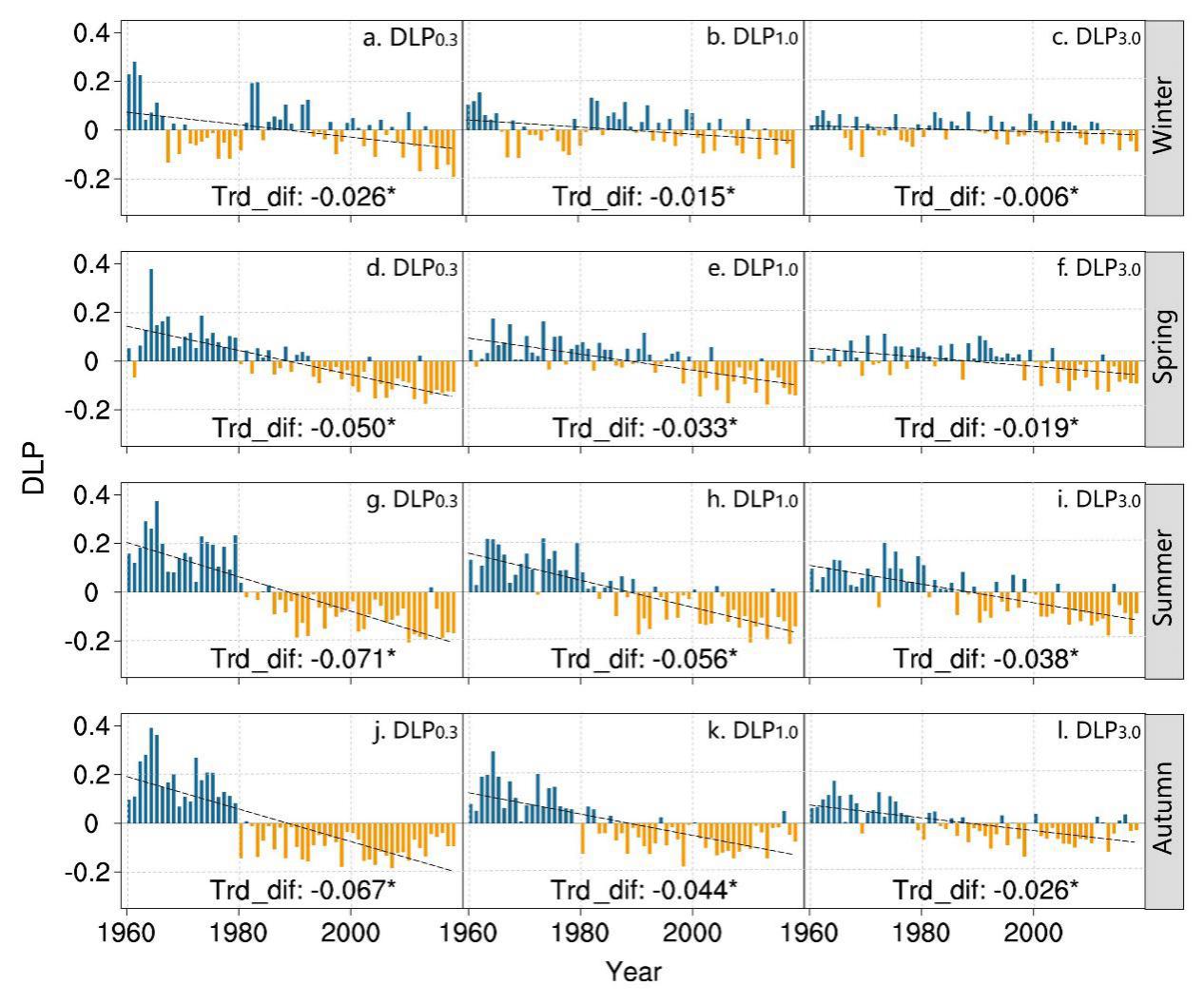


**Supplementary Figure 4.** The difference time series of the standardized anomalies of the winter **(a-c)**, spring **(d-f)**, summer **(g-i),** and autumn **(j-l)** total DLP (**a, d, g, j:** LP_0.3_; **b, e, h, k:** LP_1.0_; **c, f, i, l:** LP_3.0_) between national and rural stations in China during the period of 1960-2018. Trd_dif: the linear trend of the difference series (units: decade^-1^); asterisks: statistically significant trends at the 95% confidence level.


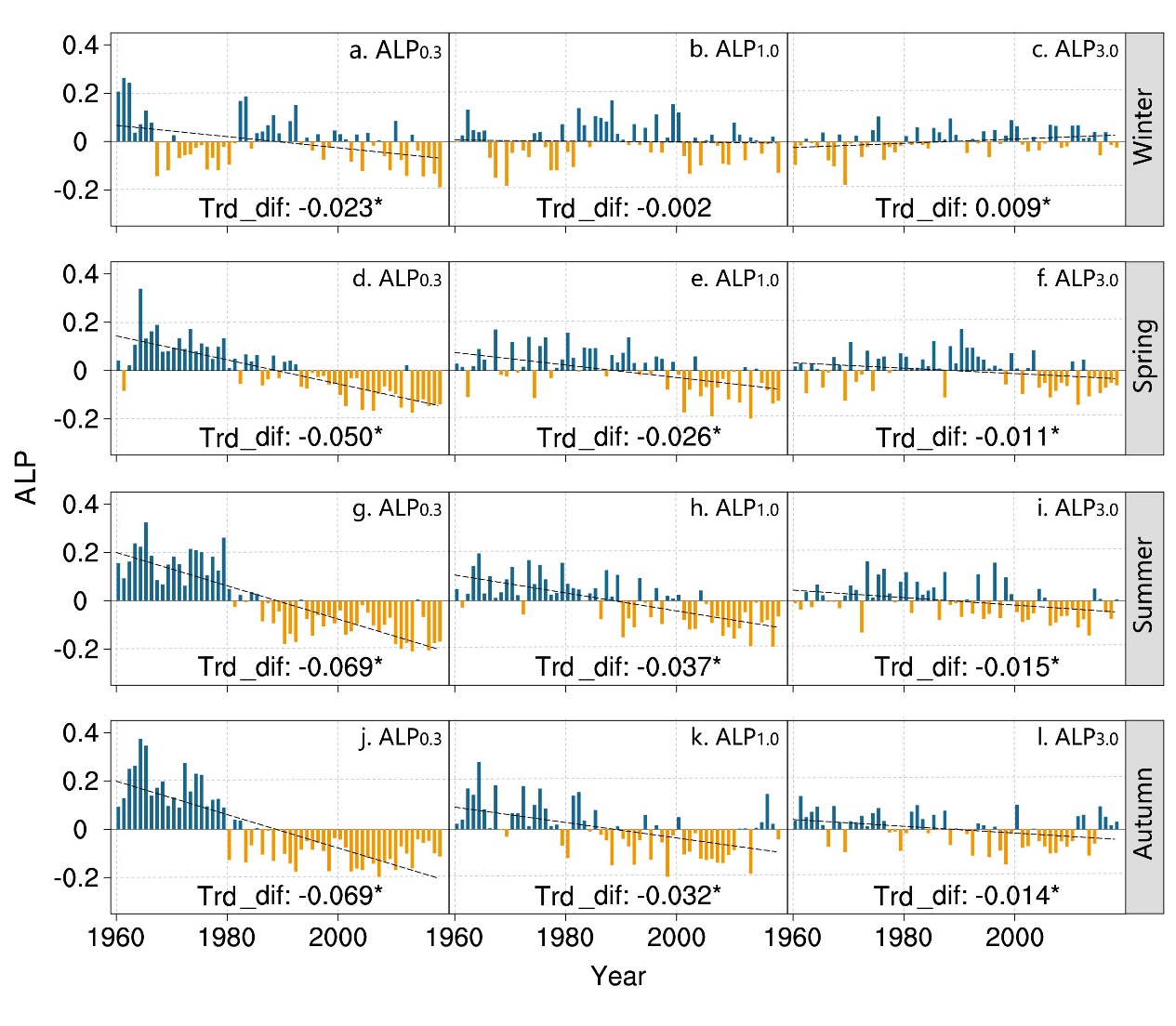


**Supplementary Figure 5.** Same as in Supplementary Figure 4 but for the ALP.


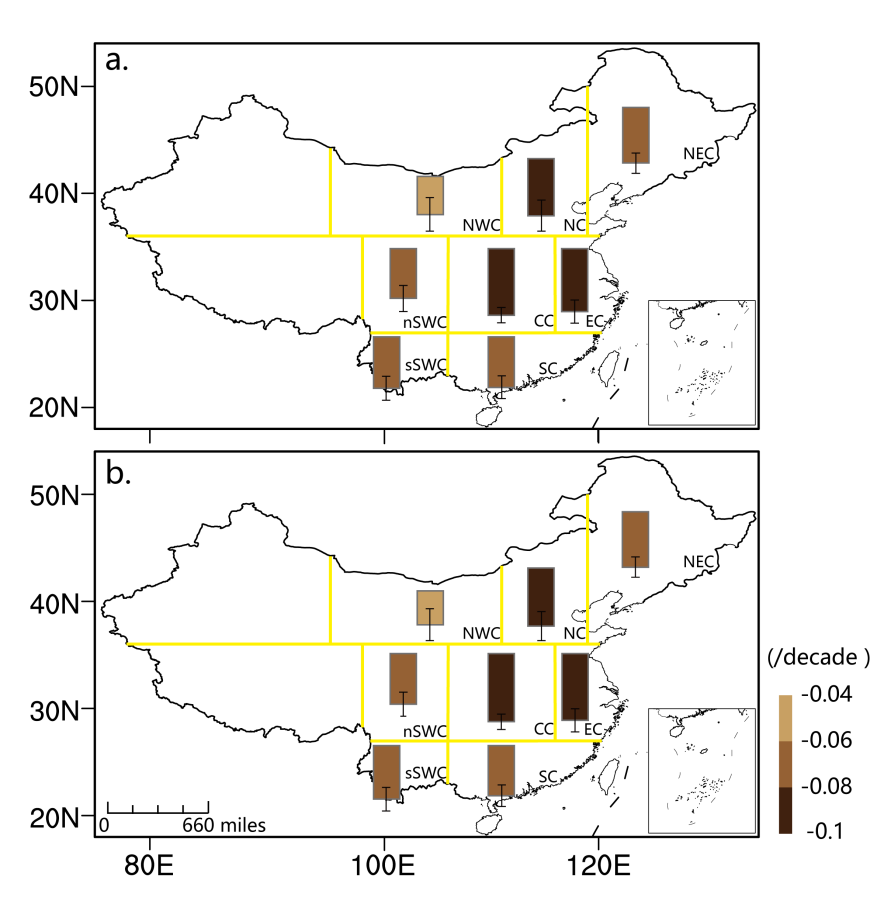


**Supplementary Figure 6.** Mean urbanization effects on the trends of the standardized anomalies of the annual total DLP_0.3_ (a) and ALP_0.3_ (b) at the national stations over eight subregions in China during the period of 1960-2018, along with their standard errors. NCAR Command Language (NCL) (Version 6.4.0; https://www.ncl.ucar.edu) was used to create the maps. Units: decade^-1^.


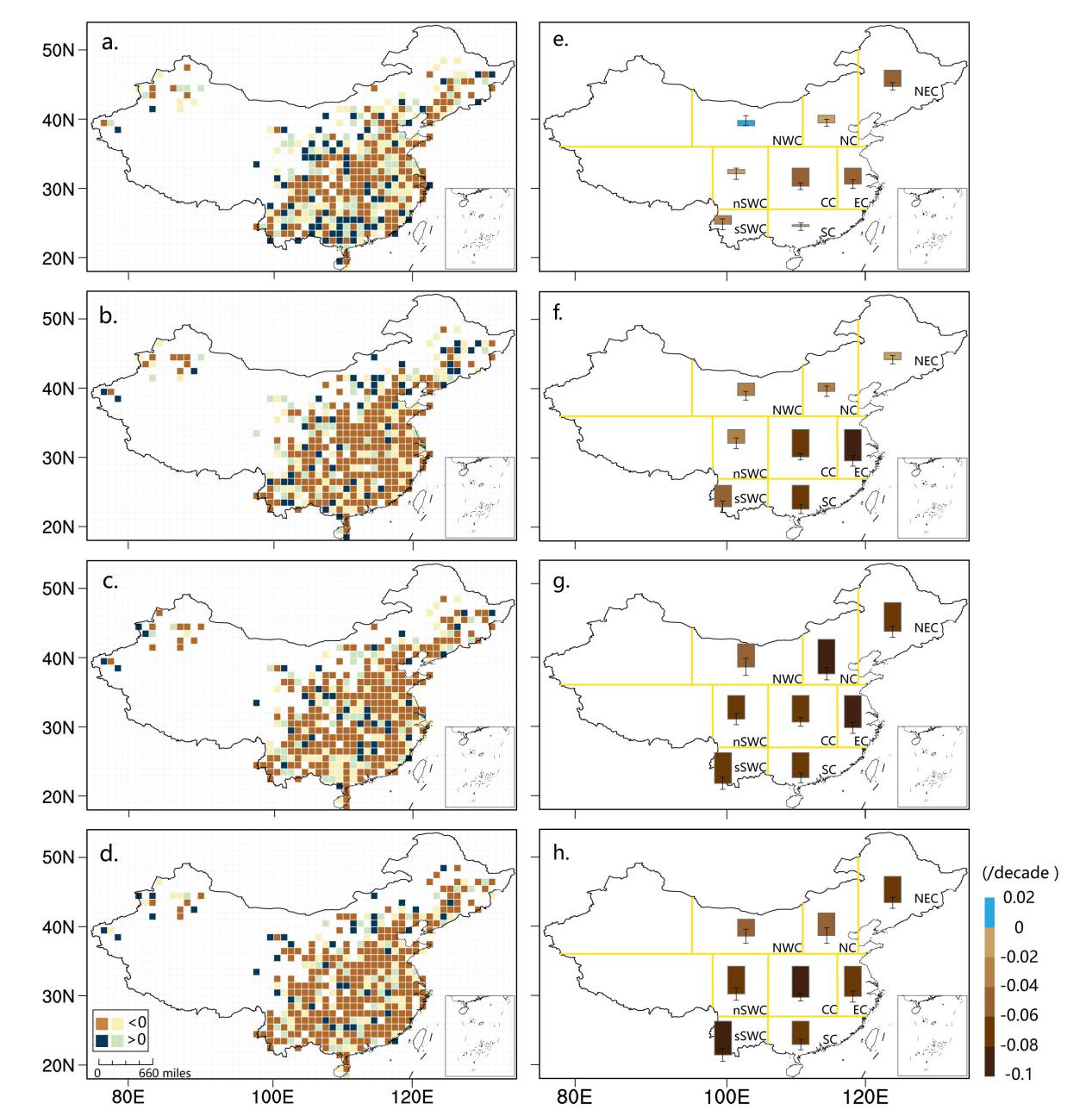


**Supplementary Figure 7.** Spatial distributions of urbanization effects on the trends of the standardized anomalies of the winter **(a)**, spring **(b)**, summer **(c),** and autumn **(d)** total DLP_0.3_ at the national stations in China during the period of 1960-2018. Grid size: 1.0°*1.0° latitude and longitude; units: decade^-1^; dark brown/blue grids: statistically significant at the 95% confidence level. Mean urbanization effects on the trends of the standardized anomalies of the winter **(e)**, spring **(f)**, summer **(g),** and autumn **(h)** total DLP_0.3_ at the national stations over eight subregions, along with their standard errors. NCAR Command Language (NCL) (Version 6.4.0; https://www.ncl.ucar.edu) was used to create the maps. Units: decade^-1^.


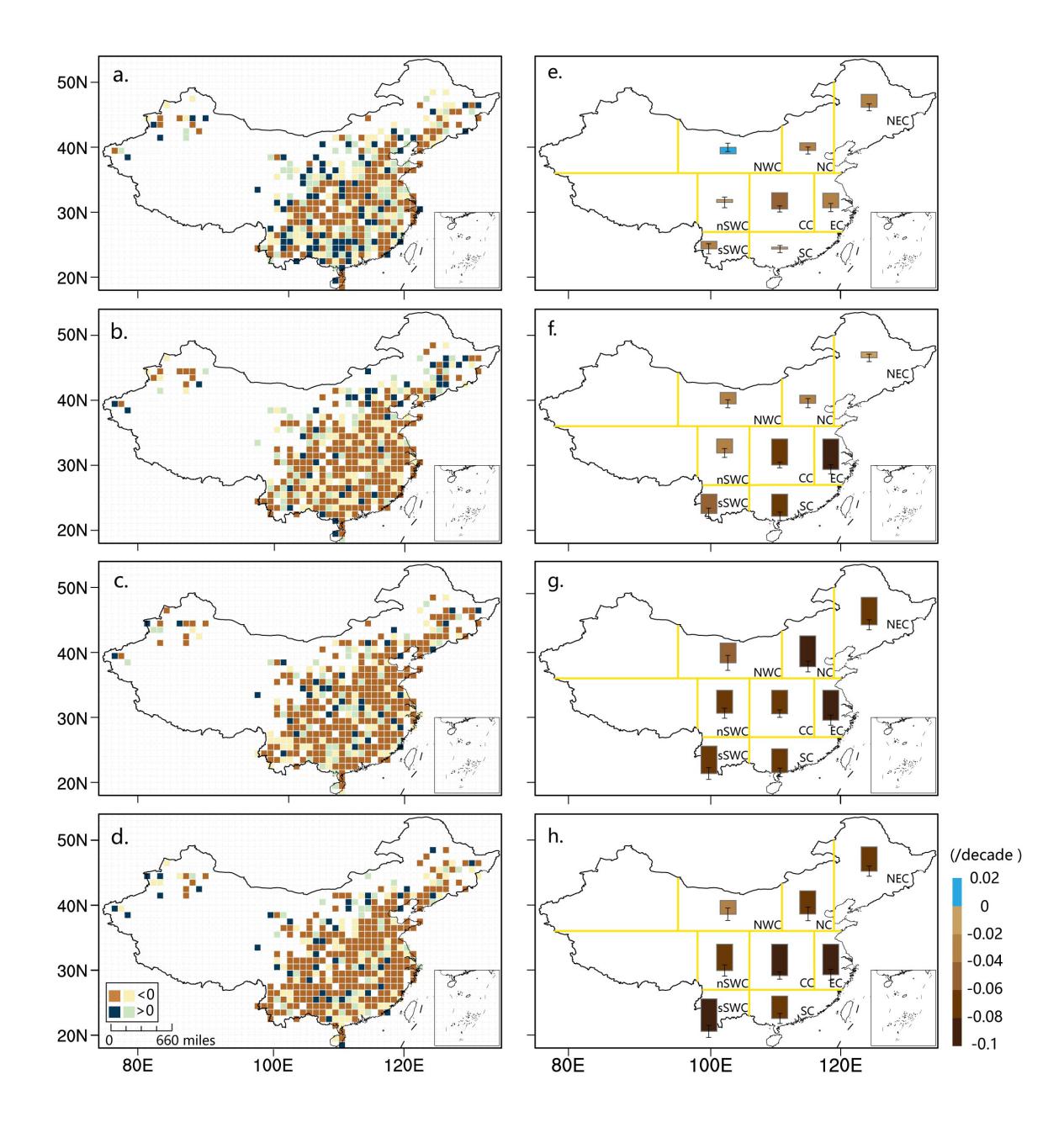


**Supplementary Figure 8.** Same as in Supplementary Figure 7 but for the ALP_0.3_.

**
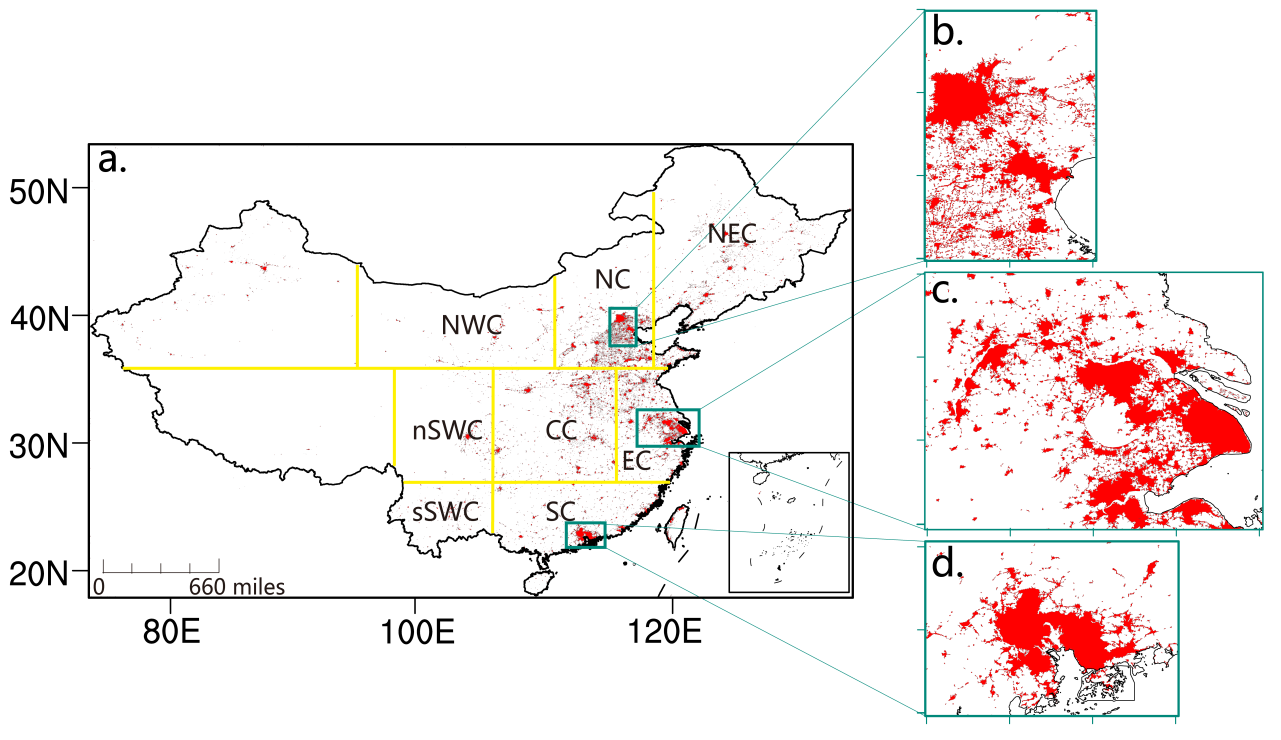


**

**Supplementary Figure 9.** Maps of artificial impervious area in China in 2018 **(a)**. Yellow lines: eight subregions in China, including northeast China (NEC), north China (NC), northwest China (NWC), east China (EC), central China (CC), northern southwest China (nSWC), southern southwest China (sSWC), and south China (SC). Green boxes: the three super-city clusters of Beijing-Tianjin-Hebei (BTH) **(b)**, Yangtze River Delta (YRD) **(c)**, and Pearl River Delta (PRD) **(d)**. NCAR Command Language (NCL) (Version 6.4.0; https://www.ncl.ucar.edu) was used to create the maps.


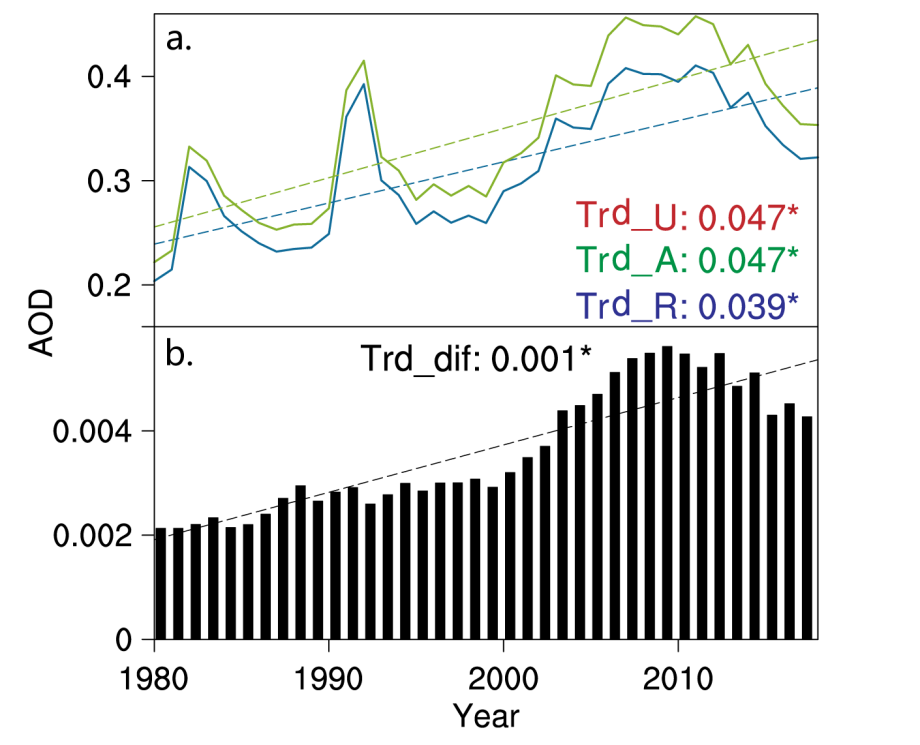


**Supplementary Figure 10.** Time series of the annual mean MERRA-2 AOD at the national stations (green lines), urban stations (red lines), and rural stations (blue lines) in China during the period of 1980-2015 **(a)** and the corresponding difference time series between national and rural stations **(b)**. Trd_A, Trd_U, and Trd_R: the linear trends of national, urban, and rural series (units: decade^-1^); Trd_dif: the linear trend of the difference series between national and rural stations (units: decade^-1^); asterisks: statistically significant trends at the 95% confidence level.


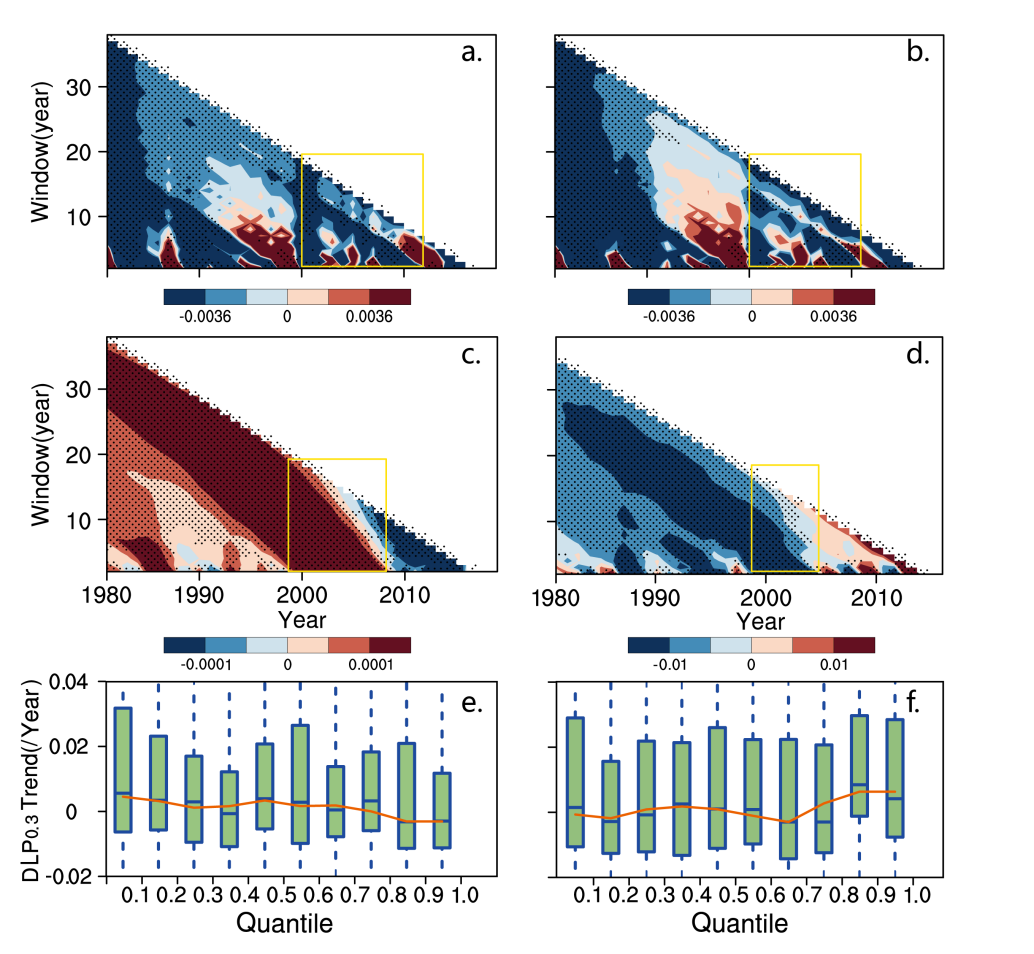


**Supplementary Figure 11.** The running trends for the urbanization effects on the trends of the standardized anomalies of the annual total DLP_0.3_ **(a)** and ALP_0.3_ **(b)**, and on the trends of the annual mean AOD **(c)** and RH **(d)** at the national stations in China during the period of 1980-2018 (colour shading in year^-1^, but %·year^-1^ for RH). The black dots: statistically significant trends at the 95% confidence level. Box-and-whisker plots of the urbanization effects on the trends of the standardized anomalies of the annual total DLP_0.3_ for different quantiles (0-0.1, 0.1-0.2, ..., 0.9-1) of the urbanization effects on the trends of annual mean AOD **(e)** and RH **(f)**. The box-and-whisker plots: the 25th percentile (top), median, and 75th percentile (bottom); the solid orange line: the mean; units: year^-1^.

**Supplementary Table 1.** Urbanization effects (left, units: decade^-1^) and urbanization contributions (right, units: %) to the trends of the standardized anomalies of the annual and seasonal total DLP_0.3_ and ALP_0.3_ at the national stations in BTH, YRD, and PRD during the period of 1960-2018. Asterisks: statistically significant trends at the 95% confidence level.

|  | | **Annual** | **Winter** | **Spring** | **Summer** | **Autumn** |
| --- | --- | --- | --- | --- | --- | --- |
| **DLP_0.3_** | **BTH** | -0.172* / 56.5 | -0.039* / 87.7 | -0.070* / 56.0 | -0.147* / 65.3 | -0.133* / 45.1 |
|  | **YRD** | -0.101* / 23.1 | -0.041* / 23.2 | -0.107* / 27.6 | -0.086* / 36.6 | -0.082* / 20.6 |
|  | **PRD** | -0.090* / 27.0 | -0.005 | -0.070* / 33.3 | -0.092* / 50.2 | -0.059* / 19.3 |
| **ALP_0.3_** | **BTH** | -0.157* / 52.8 | -0.046* / 98.2 | -0.068* / 52.9 | -0.121* / 57.5 | -0.138* / 48.2 |
|  | **YRD** | -0.103* / 23.6 | -0.033* / 19.3 | -0.107* / 28.4 | -0.092* / 41.1 | -0.081* / 20.7 |
|  | **PRD** | -0.084* / 26.6 | -0.006 | -0.059* / 30.5 | -0.094* / 53.0 | -0.059* / 20.4 |
